# Open-Label Placebo Effects on Psychological and Physical Well-Being: A Conceptual Replication Study

**DOI:** 10.32872/cpe.7679

**Published:** 2022-12-22

**Authors:** Anne-Kathrin Bräscher, Ioanna-Evangelia Ferti, Michael Witthöft

**Affiliations:** 1 Department of Clinical Psychology, Psychotherapy, and Experimental Psychopathology, Johannes Gutenberg University of Mainz, Mainz, Germany; Philipps-University of Marburg, Marburg, Germany

**Keywords:** open-label placebo (OLP) effect, expectation, brand name, personality traits, healthy sample

## Abstract

**Background:**

Contrary to traditional placebos, open-label placebos (OLP) abstain from deception, i.e., participants are openly informed to receive an inert substance. Studies in clinical and healthy samples evidence the efficacy of OLPs. This study aims to conceptually replicate and expand findings of a recent OLP study in healthy participants while implementing a within-subject design and daily instead of retrospective assessments. Additionally, the effect of a brand name on the medicine container is tested and possible predictors of the OLP effects are explored.

**Method:**

Healthy participants (N = 75) received OLP and no placebo for 5 days each (randomized sequence) and answered daily questionnaires on sleep quality, bodily symptoms, mental well-being, and psychological distress. The medicine container of half the participants had a brand name, the remaining did not. Different personality traits and situational factors were assessed.

**Results:**

Mental and physical well-being did not differ between OLP and control phase, i.e., overall, no OLP effect emerged. Contrast analysis indicated that an OLP effect emerged for sleep quality and psychological distress when no brand name was present. Further, an OLP effect emerged in persons with higher expectations for bodily symptoms (r = .23, p = .046) and psychological distress (r = .24, p = .037).

**Conclusions:**

Methodological differences to the original study are discussed as an explanation for the failure to induce overall OLP effects. Future studies should continue to replicate previous findings and determine the exact conditions of successful implementation of OLP effects in healthy as well as clinical samples.

## Highlights

- The attempt to replicate an open-label placebo effect on well-being in healthy participants failed.
- Possibly, differences in the design and time-points of assessments explain the negative findings.
- Presence of a brand name on the medicine container and possible moderators were tested.

Due to deception, the application of traditional placebos (i.e., “interventions that, owing to their intrinsic properties, are ineffective for a particular condition or symptom(s), but which may be (…) administered (…) with the aim of eliciting placebo effects”, p. 18, Blease & Annoni, 2019) in patient care can go along with ethical and legal problems as well as with a loss of trust in the therapist-patient relationship (Bundesärztekammer, 2010; Miller et al., 2005). Open-label placebos (OLP) might solve these issues since patients are openly informed about the placebo treatment, rendering deception unnecessary.

Numerous studies evidence the efficacy of OLP in different clinical contexts and two meta-analyses indicate large effect sizes (Charlesworth et al., 2017; von Wernsdorff et al., 2021). Studies in healthy participants have been conducted less frequently, although they can 1) help to shed light on underlying mechanisms of OLP effects that remain unclear to this point and 2) target primary endpoints as the improvement of well-being and physical or cognitive performance (Kleine-Borgmann et al., 2021; Saito et al., 2020). Along these lines, some studies in healthy samples explored OLP effects in experimentally induced pain (Disley et al., 2021; Kube et al., 2020; Locher et al., 2017; Schafer et al., 2015; Schneider et al., 2020; Wei et al., 2018). Few studies focused on areas other than pain perception (El Brihi et al., 2019; Guevarra et al., 2020; Kleine-Borgmann et al., 2021). Especially, El Brihi and colleagues (2019) showed that the intake of placebo pills on five subsequent days compared to not taking placebo pills can reduce psychological distress and bodily symptoms and increase mental well-being and sleep quality in healthy participants. While the dose (i.e., taking one vs. four pills each day) did not influence the OLP effects, positive expectations and adherence were significant predictors.

The primary aim of the present study was to conceptually replicate the findings of El Brihi et al. (2019) on physical and mental well-being in healthy participants. As a stricter test of the OLP effect, a within-subject design was implemented (i.e., all participants pass through a control phase without taking placebos and a placebo phase), since a control group that does not receive OLPs might be disappointed and thus artificially boost OLP effects. Further, instead of a singular retrospective assessment of relevant constructs, assessments were collected daily to avoid potential memory biases.

Knowledge on situational and personality factors that moderate OLP effects is scarce. Dispositional optimism has shown to be associated with deceptive but not open-label placebo effects (Locher et al., 2019). Yet, studies on the impact of personality factors are rare even in the investigation of deceptive placebo effects, and results tend to be inconsistent (Kern et al., 2020). Beyond that, evidence shows the influence of aspects like price, appearance, branding, and labeling on deceptive placebos (Meissner & Linde, 2018), but studies in this realm focusing on OLP effects are missing. Expanding the conceptual replication, we aimed to additionally explore whether the presence of a brand name on the medicine container would influence the OLP effect, as suggested by El Brihi and colleagues (2019), who did not vary the brand name (“placibax”) in the original study. We hypothesized that healthy participants would show OLP effects in physical and mental well-being, which would be further enhanced when the medicine container is equipped with a brand name instead of no label. Further, we exploratively assessed a range of different psychological and situational factors to potentially identify predictors of the OLP effect.

## Method

### Sample

Participants were recruited by notes on campus, social media, and e-mail distribution lists, already indicating that the study investigated the influence of placebos on well-being. In total *N* = 75 participants (*n* = 49 females, 65.3%; *M* = 32.00, *SD* = 12.75 years) were included in the study (for exclusion criteria and further information cf. Appendix A, Supplementary Materials). All participants gave their written informed consent before commencing the study. All procedures were approved by the local ethics committee (2019-JGU-psychEK-001).

### Experimental Procedure

**Figure 1:**
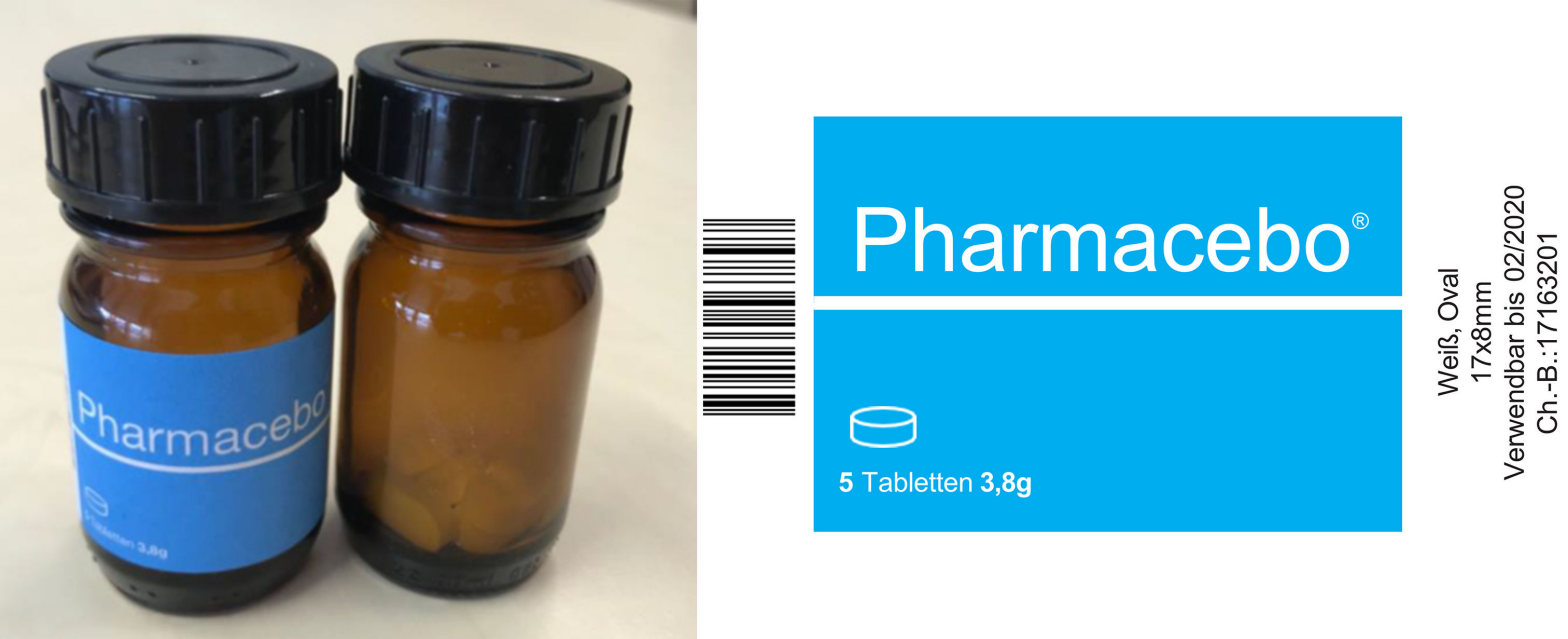
Picture of the Medicine Container *Note.* Medicine container with and without a label (left) and display of the label with the brand name (“pharmacebo”), including information on the size and weight of the pills as well as the expiration date.

In the first part of the study, participants came to the lab and filled in several psychometric questionnaires and a questionnaire on demographic information via the online platform SosciSurvey (Leiner, 2018). Suggestibility was assessed with the Creative Imagination Scale (cf. below). Subsequently, participants watched a 10-minute animated video (generated with videoscribe; cf. Appendix B, Supplementary Materials, for the narrative), addressing the four key aspects that are always communicated in OLP studies (Kaptchuk, 2018): remove the stigma of placebo effects; automatic nature of placebo responses; no requirement to believe; taking the pills is critical. The video also stressed that studies have shown beneficial effects on psychological and bodily well-being in healthy persons. After that, participant’s questions were answered and expected effects on sleep quality, bodily symptoms, mental well-being, and psychological distress were assessed using a scale from 0 (“I do not expect any effect at all”) to 10 (“I expect a very strong effect”), respectively. Finally, participants received a closed envelope containing an amber glass with five placebo pills. Half of the amber glasses (*n* = 37) had a label inscribed with “pharmacebo”, the other half of the amber glasses (*n* = 38) did not have a label (randomized; cf. Figure 1). The experimenter was blind to the kind of amber glass, which the participant received. Further, participants were informed when they should start taking the placebo pills.

The second part of the study always started on the Monday following the lab appointment, to avoid interference with weekend days. Participants either started with the placebo phase and were instructed to take a placebo every morning for five consecutive days (Monday to Friday) and then switch to the control phase (again from Monday to Friday), or they started with the control phase and switched to the placebo phase the week after. The order of placebo and control phase was randomized (random.org). During those ten days, participants received an e-mail every evening containing the link to questionnaires they were asked to fill in to assess the OLP effects as well as a question on adherence (“Did you take the placebo pill at least 6 hours ago?” yes/no). On the last day of the placebo phase, they were additionally asked how many placebo pills they had to spare. Further, on the day before the start of the placebo phase, the expected effects on all outcome measures were assessed again, using the expectancy scale.

### Outcome Measures

The following questionnaires were filled in daily. The instructions were changed where necessary to refer to the current day (instead of a longer period).

#### Warwick-Edinburgh Mental Well-Being Scale

The questionnaire (Tennant et al., 2007; German version, Lang & Bachinger, 2017) contains 14 items and assesses general mental well-being (range [14-70]). It has shown good internal consistency (α = .89 to .91), content, convergent, and discriminant validity. The retest reliability is high (*r* = 0.83, Tennant et al., 2007). The German version has shown good validity and reliability, as well (Lang & Bachinger, 2017). Internal consistency in the current study ranged between α = .91 and α = .96.

#### Profile of Mood State (POMS)

The questionnaire (McNair et al., 1971; German short version, Dalbert, 1992) assesses the current mood through 19 items. Within the present work, the subscales sorrow, hopelessness, fatigue, and positive mood (reversely coded) are summoned to build the scale psychological distress (16 items; range [16-112]). The internal consistency is high and ranges between α = .83 and .94 for the different subscales (Dalbert, 1992). The internal consistency of the scale psychological distress in the current study ranged between α = .93 and α = .96.

#### Subjective Health Complaints (SHC)

The SHC lists 29 bodily symptoms, which can be rated on an intensity scale from 0 (not at all) to 3 (severe) (rang [0-87]). It has acceptable to good internal consistency (α = .75 to .82, Eriksen et al., 1999) and is associated with healthcare utilization (Filipkowski et al., 2010). The items have been translated by the authors. Internal consistency in the current study ranged between α = .70 and α = .80.

#### Groningen Sleep Quality Scale (GSQS)

This questionnaire (Leppämäki et al., 2003; Mulder-Hajonides van der Meulen et al., 1980) contains 15 items, which can be answered with yes and no, assessing sleep quality of the previous night. Larger scores indicate poorer sleep [range 0-14]. Internal consistency was α = .88 in a sample of depressed patients (current study: α between .15 and .55).

### Measures of Psychological Factors

During the lab appointment, participants filled in the following questionnaires to assess different traits and psychological factors: State-Trait Inventory (STAI-T), NEO-Five Factor Inventory, Somatosensory Amplification Scale, Patient Health Questionnaire-15 (PHQ-15), Questionnaire on attitudes towards complementary medical treatment (QACAM). Further information on the questionnaires can be found in Appendix C, Supplementary Materials.

#### Creative Imagination Scale (CIS)

The CIS (Wilson & Barber, 1978) assesses suggestibility using standardized descriptions of ten different situations on visual, auditive, kinesthetic, and olfactory perceptions. While the experimenter reads out the descriptions, the participant is asked to imagine the situation and afterward evaluate inasmuch their imagination matched the real experience using one item for each of the ten situations. The internal consistency in the current study was α = .89.

### Statistical Analysis

Changes between the first and second assessment in expected OLP effects were tested using the Wilcoxon-signed-rank-test due to non-normally distributed data. Considering sleep quality, bodily symptoms, mental well-being, and psychological distress, respectively, as outcome variables, mixed 2x5x2-ANOVAs were performed to assess the OLP-effect (within-factor “condition”) and the influence of time (within-factor “day”) as well as brand name (between-factor “brand name”). Since the order of the phases (placebo intake or control phase in week one) did not significantly influence the results, this factor was not included in the reported analyses. Holm-corrected post hoc-tests were applied were appropriate. Contrast analyses were calculated to test the hypothesis that OLP effects were larger with a brand name. As measures of effect size, η^2^ (η^2^ ≥ 0.01 small; η^2^ ≥ 0.06 medium; η^2^ ≥ 0.14 large) and Cohen’s *d* (*d* ≥ 0.30 small, *d* ≥ 0.50 medium, *d* ≥ 0.80 large) are specified. As explorative analyses, to identify potential predictors of the OLP effect, Pearson correlations between psychological factors and the outcome measures (i.e., the difference between the average score during placebo and control phase) were calculated (*r* ≥ |.10| small; *r* ≥ |.30| medium, *r* ≥ |.50| large). The alpha level was set to 5%. Analyses were calculated with JASP version 0.14.1 (JASP Team, 2020).

## Results

### Expectation and Adherence

Adherence (i.e., intake of the placebos as instructed) was excellent. In only two instances, participants reported to have forgotten the intake once, which was confirmed by the question at the end of the OLP phase (“How many pills do you have to spare?”). Expected effects of the OLP effects were in the medium to low range of the scale and significantly decreased from the first to the second assessment (see Table 1).

**Table 1:** Expected Open-Label Placebo Effects

Outcome	Expectation after manipulation		Expectation before placebo phase		Test statistic for differences between both assessments		
	*M*	*SD*	*M*	*SD*	*W*	*p*	*r_rb_*
Sleep quality	3.41	2.99	2.82	2.85	427.5	.040	0.33
Bodily symptoms	3.73	2.95	2.88	2.76	928.00	< .001	0.58
Mental well-being	4.41	3.12	3.34	2.95	1039.50	< .001	0.63
Psychological distress	3.77	3.01	2.86	2.84	874.00	.008	0.43

*Note*. Expectations assessed at the first assessment directly after the open-label placebo manipulation and at the second assessment the day before the first intake of the open-label placebo and difference test.

### Open-Label Placebo Effects

**Figure 2:**
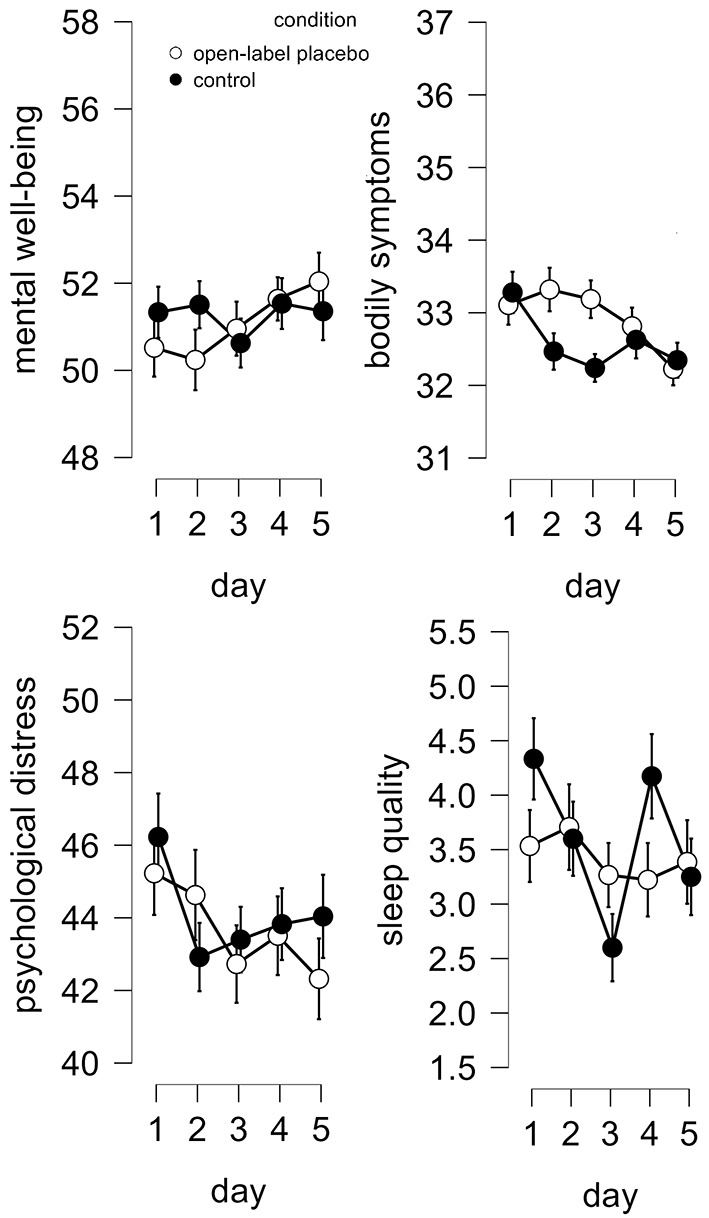
Open-Label Placebo Effects *Note.* Average scores of psychological distress, mental well-being, bodily symptoms, and sleep quality across five days each in the OLP (white) and control condition (black). Error bars represent the standard error.

Concerning sleep quality, placebo and control week did not differ significantly and this did not change over the five days, i.e., overall, no OLP effect emerged (see Table 2). Neither the main effect of day nor that of brand name were significant. A significant interaction effect between condition and brand name emerged (see Figure 2), but post hoc-tests were non-significant (all *p*s > .190). Contrary to the hypothesis, the contrast analysis showed that the difference between scores of the placebo versus the no treatment week was larger when no brand name was present, *t*(73) = -2.42, *p* = .009, indicating that a medicine container without a brand label led to an OLP effect but a medicine container without a brand label did not.

**Table 2:** Results of ANOVAs for the Respective Outcome Measures

Outcome / Factor	*df*	*F*	*p*	Effect size η^2^
Sleep quality				
Condition	1	0.43	.512	< .001
Day of the week	4	2.18	.071	.010
Condition x day	3.48	2.32	.066	.008
Label	1	0.95	.334	.004
Condition x label	1	5.85	.018	.007
Bodily symptoms				
Condition	1	1.60	.210	.002
Day of the week	3.46	4.61	.002	.006
Condition x day	3.32	2.68	.042	.004
Label	1	< 0.01	.979	< .001
Condition x label	1	< 0.01	.979	< .001
Mental well-being				
Condition	1	0.13	.716	< .001
Day of the week	3.27	1.21	.306	.002
Condition x day	3.68	0.99	.408	.001
Label	1	0.10	.749	.001
Condition x label	1	0.89	.349	< .001
Psychological distress				
Condition	1	0.16	.693	< .001
Day of the week	3.51	2.50	.051	.003
Condition x day	3.68	0.71	.572	.001
Label	1	0.01	.910	.001
Condition x label	1	3.96	.050	.001

With regards to bodily symptoms, placebo and control week did not differ significantly (see Table 2). A significant interaction effect between condition and day emerged (see Figure 2), but post hoc-tests were non-significant (all *p* > .240). The five days differed significantly for reported bodily symptoms and post-hoc tests indicated that bodily symptoms decreased when comparing Day 1 to Day 5, *t*(74) = 4.11, *p* < .001, *d* = 0.48, remaining post hoc-tests all *p* > .056. Bodily symptoms did not differ significantly depending on the presence of a brand name and no significant interaction emerged between brand name and condition. The contrast analysis did not point to a differential effect depending on the presence of a brand name, *t*(73) = -0.03, *p* = .490.

Placebo and control week did not differ significantly concerning mental well-being (see Table 1, Figure 2) and this did not change over the five days, i.e., no overall OLP effect emerged. Neither the main effect of day nor of label, nor the interaction effect between label and condition reached significance. The contrast analysis did not point to a differential effect depending on the presence of a brand name, *t*(73) = -0.94, *p* = .175.

For psychological distress, similarly, placebo and control week did not differ significantly (see Table 2, Figure 2) and no significant interaction effect between condition and day emerged. No main effect of day and label reached significance. The interaction effect between brand name and condition just reached significance, but post hoc-tests were non-significant (all *p*s > .561). Contrary to the hypothesis, the contrast analysis indicated that the difference between scores of the placebo versus the no treatment week was larger without the brand name, *t*(73) = -1.99, *p* = .025, indicating that a medicine container without a brand label led to an OLP effect but a medicine container without a brand label did not.

### Identification of Predictors

Expectation assessed the day before the placebo intake (2^nd^ assessment) significantly correlated with the difference between scores taken in the placebo versus the no treatment week for the outcome measures bodily symptoms (*r* = .23, *p* = .046) and psychological distress (*r* = .24, *p* = .037), respectively. The effect sizes of the remaining correlations with other psychological factors were partly in the small range but did not reach significance (Suppl. Table 1 in Appendix C, Supplementary Materials).

## Discussion

This study aimed to conceptually replicate findings of a previous experiment (El Brihi et al., 2019) that demonstrated small to medium OLP effects (*d* = 0.28-0.50) on mental and physical well-being in healthy participants. Using a within-subject design and daily assessed sleep quality, bodily symptoms, mental well-being, and psychological distress, overall no significant OLP effect emerged in the present study. Other than hypothesized, a brand name on the medicine container hindered OLP effects in sleep quality and psychological distress. Explorative analyses hinted at expectation as a possible predictor of the OLP effects in bodily symptoms and psychological distress.

Several reasons might explain the failure to replicate the results of the original study. General issues refer to possible differences in the populations investigated (e.g., language, country, ethnicity, etc.). It is also possible that floor or ceiling effects prevented the development of OLP effects in this healthy sample, yet a comparison to normative values is hardly possible due to altered instructions (referring to the last day instead or a week or else). Further, the present study partly used other outcome measures than the original study (POMS, GSQS). The two most important differences to the original study refer to the design of the studies and the time points of assessment. Employing a within-subjects design has the advantage that every participant serves as their control group, i.e., no random differences will confound the effects of interest. This is especially important since concerns regarding the control group in OLP studies have been voiced (Blease et al., 2020). It can be speculated that the control group in the original study was less motivated or did not pay as much attention to the symptoms in question as the group that received placebos because they were neither reminded to attend to possible effects by taking a pill nor by filling in daily questionnaires, which might have artificially boosted OLP effects. Regarding the time points of assessment, the present study assessed symptoms daily, while the original study assessed symptoms once after five days of placebo intake or control phase. This retrospective assessment might have led to an overestimated OLP effect due to memory biases (Ebner-Priemer & Trull, 2009). Another potential reason for the non-existent OLP effects might be the mode of presentation of the information concerning OLP effects to the participants. To standardize this aspect of the study, participants watched an animated video that conveyed the relevant information. In other OLP studies, this information is given in a conversation between the experimenter and the participant. Research indicates that (open-label) placebo effects benefit from trustworthy, friendly and empathetic treatment providers (Gaab et al., 2019; Kube et al., 2021). Possibly, the therapeutic alliance between treatment provider and participant was adversely affected by implementation of the video instead of personal communication in the present study. Feasibly, participants in our study were not as attentive or engaged or the video just was less convincing than a personal conversation. Along these lines, expected OLP effects were somewhat lower in our study (range of *M* = 3.4 and *M* = 4.4) compared to the original study (*M* = 4.9). Interestingly, a recent study (Kube et al., 2021) failed to find OLP effects in allergic rhinitis when information on OLP was conveyed in an online setting. This result emphasizes the importance of the mode of presentation.

Although many previous studies evidence OLP effects in clinical (Carvalho et al., 2016; Charlesworth et al., 2017; von Wernsdorff et al., 2021) as well as healthy samples, including those on mental and physical well-being (El Brihi et al., 2019; Guevarra et al., 2020; Kleine-Borgmann et al., 2021), some studies were only partly successful (context of itch, Meeuwis et al., 2019; Meeuwis et al., 2018) or failed to induce OLP effects, e.g., in chronic back pain (Ikemoto et al., 2020), nausea (Barnes et al., 2019), wound healing (Mathur et al., 2018), and allergic rhinitis (Kube et al., 2021). Future studies should find out, whether OLP effects can be reliably induced in healthy participants and which conditions are key.

We hypothesized that a brand name on the medicine container would increase the OLP effect because usually medication is labeled and in deceptive placebos, brand names lead to larger effects (Meissner & Linde, 2018). However, contrary to that, the difference in scores between placebo and control week tended to be increased when no brand name was present for two of the outcome measures, namely psychological distress and sleep quality, while the presence of a brand name did not influence the effects of the two remaining outcome measures. Possibly, when reading the label “pharmacebo”, participants were reminded that they are about to take a placebo, which might have counteracted conditioned effects based on previous experiences with medication. It would be worthwhile to replicate the present findings and to investigate the effect of a brand name that does not hint at the placebo context in future studies.

Several possible predictors of OLP effects were explored. Suggestibility, neuroticism, extraversion, openness, conscientiousness, habitual anxiety, somatization, somatosensory amplification, and a positive attitude towards CAM or conventional medicine were not significantly associated with the difference in scores of the placebo and control week. These findings are similar to those of a study on experimental heat pain in healthy participants that did not find associations of the OLP effect with optimism, pessimism, openness, locus of control, and positive attitudes towards CAM (Locher et al., 2019). Interestingly, relevant traits in the context of deceptive placebo effects do not necessarily play a role in OLP effects (cf. optimism, Locher et al., 2019). Thus, more research is needed to identify facilitating personality traits of OLP effects, should they exist.

In line with our assumptions, expectations were a significant predictor for the OLP effects in bodily symptoms and psychological distress. Results of previous studies concerning the role of expectations are inconsistent; whereas some studies showed a relationship between OLP effect and measures of expectation (El Brihi et al., 2019, not for sleep quality, however; Kleine-Borgmann et al., 2021) other did not (Guevarra et al., 2020; Kube et al., 2021). Possibly, the time point of assessment of the expectations is an important aspect to consider. In the present study, participants expressed higher expectations directly after the information about OLP effects and expectations significantly decreased in the second assessment before the placebo phase. Yet, only expectations of the second assessment were significantly associated with the OLP effects. Therefore, possibilities should be explored that keep expectations stable for a longer period of time, for example sending patients written information on the open-label placebo effect to boost expectations right before the intake of the placebos.

Some limitations need to be mentioned for the present study. The animated video was meant to increase standardization when giving participants information about OLP effects. It would have been helpful to validate the animated video in a pilot study, test whether the information was conveyed as desired and whether alliance would be affected. Since the placebo and control phases of the study took place in the field instead of in a controlled lab environment, we cannot be sure whether participants took the placebos as prescribed. Yet, this approach has higher ecological validity than most OLP studies that comprise healthy participants, as it closely resembles realistic conditions (i.e., taking medication at home). Further, besides asking about the intake of the placebo pills daily, we confirmed the participants’ statements by asking how many pills they had to spare at the end of the study. The employed brand name “pharmacebo” might have not been optimally chosen, since it includes the term “pharma” and thus could be misleading. Yet, the results do not support this notion as participants whose medicine container did not have a label tended to benefit better from the placebos. It would be helpful to investigate the impact of different brand names and their connotations in a future study. Finally, analyses were based solely on self-report data. Assessing objective data, for example with the help of fitness watches tracking sleep parameters, could be a beneficial addition.

To conclude, open-label placebo effects are a promising phenomenon that has the potential to improve patient care while respecting patients’ autonomy. Similar to other recent investigations, this study failed to find overall OLP effects in mental and physical well-being in healthy participants. It will be important to continue replicating previous findings and to determine the exact conditions of successful implementation of OLP effects in healthy as well as clinical samples.

## Supplementary Materials

The Supplementary Material contains further information on the sample, the manualized narrative provided in the animated video, further information on questionnaires assessed, and a supplementary table with correlations of the difference between scores taken in the placebo versus the no treatment week of the outcome measures with psychological factors (for access see Index of Supplementary Materials below).



BräscherA.
FertiI.
WitthöftM.
 (2022). Supplementary materials to "Open-label placebo effects on psychological and physical well-being: A conceptual replication study"
[Additional information]. PsychOpen. 10.23668/psycharchives.12173
PMC988112336762351
